# Sex differences in blood pressure control in SHR: lack of a role for EETs

**DOI:** 10.14814/phy2.12022

**Published:** 2014-05-20

**Authors:** Mohadetheh Moulana, Karen Hosick, James Stanford, Huimin Zhang, Richard J. Roman, Jane F. Reckelhoff

**Affiliations:** 1 Department of Psychiatry, University of Mississippi Medical Center, Jackson, Mississippi; 2 Women's Health Research Center, University of Mississippi Medical Center, Jackson, Mississippi; 3 Department of Physiology and Biophysics, University of Mississippi Medical Center, Jackson, Mississippi; 4 Department of Pharmacology and Toxicology, University of Mississippi Medical Center, Jackson, Mississippi

**Keywords:** EETs, female, kidney, renal vasculature

## Abstract

The mechanisms responsible for the gender difference in blood pressure (BP) in humans are not clear. Over the past several years we have studied the spontaneously hypertensive rat (SHR) as a model of sex differences in BP control. In the present study, we tested the hypothesis that renal vascular and microsomal epoxyeicosatrienoic acid (EET) levels are higher in females than males, and increasing vascular EETs by blocking epoxide hydrolase with AUDA will reduce BP more in males than females. Renal vascular and microsomal EETs were higher in female SHR than males. Mean arterial pressure (MAP by telemetry) was higher in males than females during the baseline period of 6 days, and although the epoxide hydrolase inhibitor, AUDA, given for 10 days increased renal microvascular EETs in both groups, AUDA did not affect MAP in either group. These data suggest that EETs do not contribute to the sex differences in hypertension in young SHR.

## Abstract (precis)

e12022

Levels of vasodilator EETs are higher in renal microvasculature of female SHR than males. Blockade of epoxide hydrolase that increases EETs has no effect on blood pressure in either male or female SHR. The data suggest that lack of EETs do not contribute to hypertension in either male or female SHR.

## Introduction

Men typically have higher blood pressure (BP) than do women until after menopause as determined by ambulatory BP monitoring studies (Lima et al. 2012). Over the last several years we have used the spontaneously hypertensive rat (SHR) as a model for sex differences in BP as male SHR have higher BP than do females (Reckelhoff et al. 1998). We have studied numerous prohypertensive systems to evaluate the potential roles these systems may play in mediating the sex differences in BP control in SHRs.

In studying arachidonic acid metabolites, we found that thromboxane receptor antagonism reduces BP in male SHR, but not females, despite the fact that females express a higher level of thromboxane B2 in their urine than males (Acosta Cazal et al. 2004). In addition, thromboxane synthesis inhibition did not reduce BP in either male or female SHR, suggesting that thromboxane receptor contributes to the sex difference in BP but not thromboxane itself.

The roles played by other arachidonic acid metabolites have also been evaluated in SHR. For example, 20‐hydroxyeicosatetraenoic acid (20‐HETE) levels are increased in male SHR compared to WKY controls (Sacerdoti et al. 1989; Omata et al. 1992). However, 20‐HETE can act as both a prohypertensive because it causes vasoconstriction of the preglomerular microvasculature and an antihypertensive because it can decrease tubular sodium reabsorption (Roman 2002). Su et al. (1998) reported that blockade of 20‐HETE synthesis reduces BP in young male SHR. However, we found that 20‐HETE synthesis blockade failed to reduce the BP in young females (Yanes et al. 2011). In contrast, we found that 20‐HETE blockade did reduce the BP in old female SHR that exhibit significant exacerbation of hypertension with aging such that BPs are either similar to or higher than in males (Yanes and Reckelhoff 2011; Yanes et al. 2011).

Another arachidonic acid metabolite that may contribute to the sex difference in BP control in SHR is epoxyeicosatrienoic acids (EETs). EETs act as an endothelium‐dependent hyperpolarizing factor on the renal microcirculation (Imig 2013), thus acting as vasodilators. In addition, EETs activate renal microvascular smooth muscle cell large conductance calcium‐activated potassium channels (K_Ca_). EETs are synthesized by epoxygenase and metabolized to DiHETEs by epoxide hydrolase (sEH). There is evidence indicating that epoxide hydrolase (EH) activity is elevated in male SHR compared to WKY (Harris and Hammock 2013), and thus the renal levels of EETs are markedly reduced. Studies have not been done in female SHR, and thus little information is available as to whether sex differences in the expression of EETs may contribute to the sex differences in BP in SHR.

Given the lack of information regarding potential sex differences in EETs in the kidney of SHR, we measured the levels of EETs in renal microvasculature and microsomal fractions and determined whether inhibition of EH would affect BP in young male and female SHR. We tested the hypothesis that females would have higher levels of EETs based on the fact that their BPs are lower and that increasing EETs by inhibiting EH would cause a greater reduction in BP in males than females.

## Methods

### Rats

Experiments were performed on male and female SHR (aged 12 weeks, *n* = 16 males, 16 females) that were bred in the Laboratory Animal Facility (LAF) of the University of Mississippi Medical Center from stock originally obtained from Taconic Laboratories (Hudson, NY). Rats were maintained on standard laboratory chow (Teklad, Harlan Sprague Dawley, Indianapolis, IN) and tap water with 12 h:12 h light:dark cycle. All protocols were reviewed and approved by the Institutional Animal Care and Use Committee of the University of Mississippi Medical Center, and studies were performed in accordance with the Guide for the Care and Use of Laboratory Animals, 8th Edition, 2011, National Institutes of Health.

### Inhibition of epoxide hydrolase

The EH inhibitor, 12‐(3‐adamantan‐1‐yl‐ureido)‐dodecanoic acid (AUDA 25 mg in 0.075% ethanol/0.05% *β*‐cyclodextrin/L; Cayman Chemical, Ann Arbor, MI), was given in drinking water (Imig et al. 2005). Water intake of the animals was monitored and the concentration of the drug in the drinking water was adjusted daily to deliver an average dose of 2.1–2.7 mg/kg body weight/day. Control rats received the vehicle (ethanol/*β*‐cyclodextrin) in drinking water.

### Telemetry

Radiotelemetry transmitters (Data Sciences International, New Brighton, MN) were implanted into the abdominal aorta of rats (12 weeks of age), as previously described (Yanes et al. 2011). Two weeks after implantation, telemetry transmitters were turned on, and mean arterial pressure (MAP) was measured 24 h per day during a 6‐day baseline period and then AUDA was added to the drinking water, and MAP was measured 24 h per day for an additional 10 days.

### Measurement of EETs

The endogenous concentrations of EETs (14,15‐, 11,12‐, 8,9‐, and 5,6‐EETs) and DiHETEs (metabolite of EETs) were assessed in renal microvessels and microsomal fractions from untreated young male and female SHR (*n* = 4/group), as previously described (Olearczyk et al. 2009). Renal vessels were isolated using the Evans blue sieving method and homogenized in 1 mL of ice cold PSS, and renal microsomal fractions were isolated and centrifuged, as we previously described (Yanes et al. 2011). The samples were extracted twice with three volumes of ethyl acetate, and the concentrations of eicosanoids were measured using liquid chromatography/mass spectroscopy (LC/MS/MS). In rats that were given AUDA or vehicle treatment (*n* = 6/group each for control males or females and treated males or females), EETs and DiHETEs were measured in renal microvessels only.

### Statistical analyses

Data are presented as mean ± SEM. Statistical analyses were performed with SigmaPlot v11 (Systat Software, Inc., San Jose, CA). MAP changes between young male and female SHR during baseline, and with and without AUDA were analyzed using a repeated measure analysis of variance (ANOVA) followed by Student–Newman–Keuls post hoc comparisons. Differences in renal vascular and microsomal EETs and DiHETEs were done by ANOVA as well. A *P* < 0.05 was considered statistically significant.

## Results

### Sex differences in renal EETs and DiHETEs in untreated SHR

Renal vascular EETs were slightly higher in females than males (5.08 ± 0.70 vs. 3.36 ± 0.15 pmol/mg; *P* < 0.05). Renal microsomal EETs were 4.5‐fold higher in females than males (642.38 ± 9.82 vs. 140.78 ± 10.64 pmol/mg; *P* < 0.001).

### Effect of EH inhibition in male and female SHR

**Figure 1.**
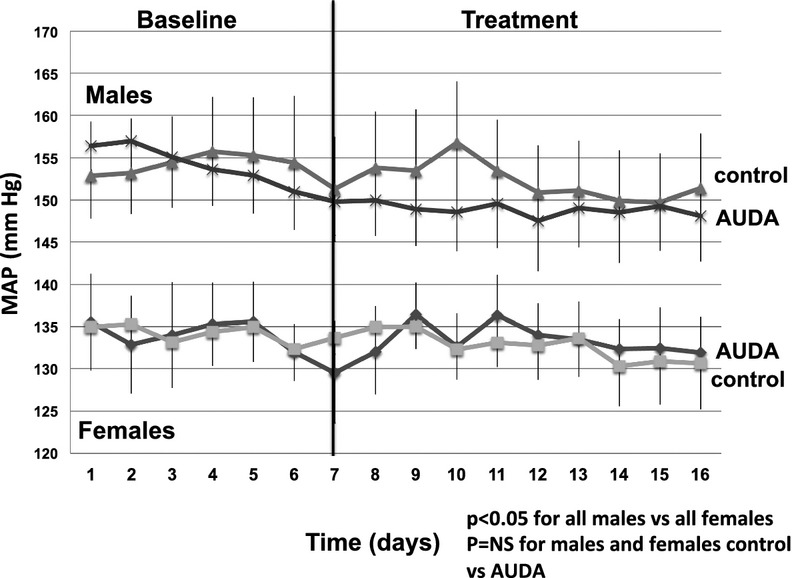
Blood pressure was higher in young male SHR than females, and chronic AUDA, soluble epoxide hydrolase inhibitor, had no effect on mean arterial pressure (MAP) in either group. MAP was measured for 6 days during the baseline period and then AUDA was given in the drinking water for 10 days as described in Methods.

**Figure 2.**
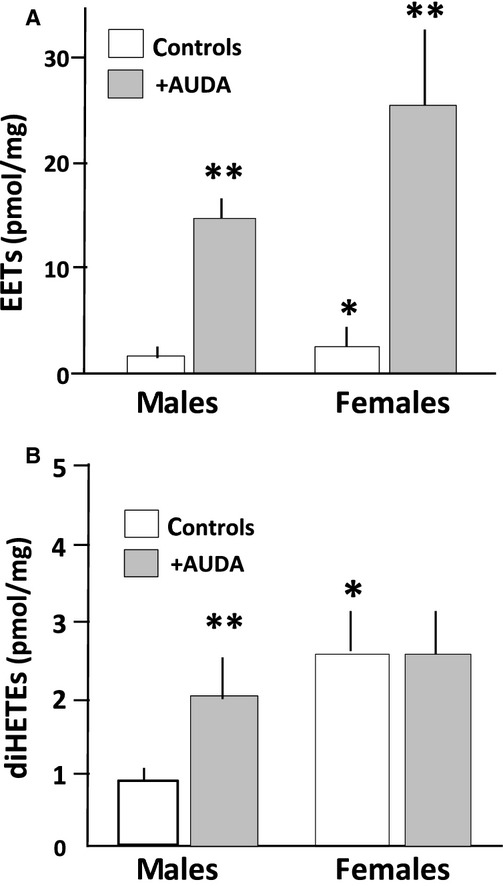
Renal microvascular (A) EETs and (B) DiHETEs in control and AUDA‐treated young male and female SHR. Rats were treated for 10 days with AUDA, soluble epoxide hydrolase inhibitor, and at the end of the study renal microvessels were isolated for measurement of EETs and DiHETEs by LC/MS/MS as described in Methods. *P < 0.05 control females versus control males; **P < 0.05, AUDA‐treated versus control animals of either sex.

Mean arterial pressure during the baseline period was significantly higher in male SHR than females (Fig. 1), and treatment with AUDA had no effect on MAP in either males or females. Renal microvascular EET levels were slightly higher in control females than males, and AUDA increased EETs in both groups (Fig. 2A), more in females than males. DiHETE levels were also significantly higher in control females than males, and AUDA increased diHETEs in males but not in females (Fig. 2B).

## Discussion

In the present study we found that EETs are higher in renal microvasculature and microsomal fractions in females than males, but that increasing EETs to higher levels does not impact BP in either group. These studies mark a continuation of our previous studies to evaluate the roles arachidonic acid metabolites play, if any, in mediating the sex differences in hypertension in SHR.

The endothelium‐dependent hyperpolarizing effect of EETs on the renal microcirculation is estrogen and estrogen receptor dependent via PI3K/Akt pathways (Harris and Hammock 2013). We have shown previously that ovariectomy of young female SHR has no effect on their BP (Reckelhoff et al. 1998, 2000). Thus it may not be surprising that increasing EETs with AUDA had no depressor effect in female SHR. However, because we have not specifically blocked synthesis of EETs in females, we cannot rule out the possibility that the higher levels of EETs could contribute to their lower BP. The fact that DiHETEs are also significantly higher in the renal microvasculature of females suggests that EH levels are also higher in females than males thus contributing to increased metabolism of EETs. This concept is supported by the significantly greater increase in EETs, but not DiHETES in females treated with AUDA. Incidentally, in contrast to our data, there is evidence that treatment of SHR dams with AUDA from 1‐week postgestation until weaning at 4 weeks of age, reduced BP in female offspring at 28–30 weeks of age by approximately 10 mmHg, but had no effect in male offspring (Koeners et al. 2011). In contrast, when rats were treated with AUDA beginning at 8 weeks of age, there was no effect on the BP in females at 12 weeks of age. Taken with the present study, these data suggest that EETs may contribute to the development of hypertension early in the nephrogenic period in females only.

In addition to the interaction between EETs and estrogens, there is also evidence that there are interactions between androgens and EETs. For example, male and female mice that are null for soluble EH (Ephx2) have increased EET:DiHETE ratios, and the males have decreased plasma testosterone levels (Luria et al. 2009). In support of this interaction between androgens and EETs, Liu et al. (2012) found that dihydrotestosterone treatment of cytochrome P450 4F2 transgenic mice caused a significant reduction in renal EETs and an increase in BP. In young male SHR, testosterone levels are elevated, and in fact, the hypertension is androgen‐mediated as gonadal removal in males reduces BP to levels found in females (Reckelhoff et al. 1998). Thus it is not surprising that EET levels would be reduced in male SHR. However, it is not clear why increasing EETs in males failed to reduce their BP.

The data from this study add to our knowledge regarding the potential mechanisms responsible for hypertension in male and female SHR. We have shown in other studies that the renin–angiotensin system (RAS) plays a significant role in mediating the hypertension in both male and female SHR. Blockade of the RAS with either angiotensin‐converting enzyme inhibitors or angiotensin AT1 receptor antagonists reduces BP to approximately 110 mmHg in both males and females (Reckelhoff et al. 2000). Furthermore, blockade of the RAS prevents androgens from being able to increase BP in males, thus suggesting that androgens increase BP via activation of the RAS. In support of this concept, we have shown that androgens upregulate renal angiotensinogen in male SHR and other models. Interestingly, Imig et al. (2002) reported that blockade of EH with AUDA prevented angiotensin II hypertension. As the RAS plays such a profound role in controlling BP in both male and female SHR, it is surprising then that increasing EETs with AUDA did not alter BP in either sex.

In addition to lack of contribution of EETs to hypertension in young SHR, we and others have shown that endothelin ET_A_ receptor (ET_A_R) antagonism plays no role in the hypertension in either males or young females (Deng and Schiffrin 1998; Yanes et al. 2005). However, ET_A_R antagonism does reduce BP modestly in old female SHR again emphasizing the differences in mechanisms of BP control with aging.

Finally, we have shown in the past that sympathetic nervous system and the renal nerves contribute to hypertension in both male and female SHR (Iliescu et al. 2006; Maranon et al. 2014). However, there is a sex difference in the mechanisms responsible for sympathetic activation. Pro‐opiomelanocortin via its melanocortin‐4 receptor (MC4R) is responsible for increasing sympathetic nervous system activity leading to increase in BP and increased thermogenesis (Hall et al. 2010). da Silva et al. (2008) reported that intracerebroventricular infusion of the MC4R antagonist reduces BP in young male SHR. However, when we recently evaluated this as a potential mechanism in female SHR, we found that MC4R antagonists had no effect on BP in either young or old female SHR, but did reduce the BP in old males (Maranon et al. 2014). These data show that while sympathetic activation is important in mediating the hypertension in both male and female SHR, the mechanisms responsible for sympathetic activation are different between the sexes.

## Conclusions

**Figure 3.**
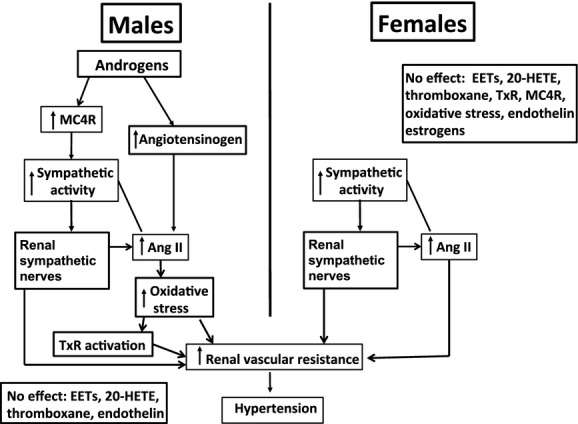
Schematic diagrams of the mechanisms responsible for hypertension in male and female SHR. MC4R, melanocortin‐4‐ receptor; Ang II, angiotensin II; TxR, thromboxane receptor.

As shown in Figure 3, the mechanisms responsible for hypertension in male and female SHR are somewhat but not completely similar. In males the hypertension is mediated by androgen‐mediated activation of the RAS that is likely enhanced by the sympathetic nervous system and the renal nerves. Activation of the sympathetic nervous system is mediated at least in part by androgen‐mediated activation of the MC4R (Maranon et al. 2014). Previous studies have shown that oxidative stress and thromboxane receptors (TxR) (perhaps activated by oxidant F2‐isoprostanes; Reckelhoff and Romero 2003) contribute to the hypertension in young males. Oxidative stress may also be increased by RAS activation in males. Hypertension in males is not mediated by 20‐HETE, EETs, or endothelin. In contrast, in female SHR, estrogens do not contribute to the hypertension (Reckelhoff et al. 1998), but the sympathetic nervous system, renal nerves, and the RAS do contribute (Yanes and Reckelhoff 2011), although sympathetic activation is not mediated by MC4R activation (Maranon et al. 2014). In females prohypertensive mediators that do not contribute to hypertension in female SHR include oxidative stress, endothelin, thromboxane or TxR, or 20‐HETE (Acosta Cazal et al. 2004; Sartori‐Valinotti et al. 2007; Yanes et al. 2011; Yanes and Reckelhoff 2011). Although not fully discussed, the differences in mechanisms responsible for hypertension in male and female SHR are multiplied with aging (Yanes and Reckelhoff 2011; Lima et al. 2012).

In conclusion, hypertension in women has been shown to be less well controlled than in men. Future studies should be done to evaluate differences in prohypertensive systems in humans in order to develop better patient‐based therapeutics for men and women.

## Conflict of Interest

None declared.
